# Metabolizable energy and standardized ileal amino acid digestibility of full-fat soybeans for broilers are influenced by wet-heating, expansion temperature, and autoclaving time

**DOI:** 10.1016/j.psj.2022.102016

**Published:** 2022-06-20

**Authors:** M.R. Abdollahi, M. Wiltafsky-Martin, F. Zaefarian, V. Ravindran

**Affiliations:** ⁎ Monogastric Research Centre, School of Agriculture and Environment, Massey University, Palmerston North 4442, New Zealand; † Evonik Operations GmbH, Animal Nutrition, 63457 Hanau, Germany

**Keywords:** amino acids, broiler, digestibility, full-fat soybean, heat-treatment

## Abstract

The influence of wet-heating (**WH**) and expansion temperature (**ET**), and autoclaving time (**AT**) on the nitrogen-corrected apparent metabolizable energy (**AME_n_**) and standardized ileal digestibility (**SID**) of AA in full-fat soybeans (**FFSB**) for broilers was examined in 2 experiments. The AME_n_ and SID AA of FFSB were determined by the difference and direct methods, respectively. In Experiment 1, raw FFSB (K0) were either treated by WH at 80°C for 1 min and expanded at 115°C (K1-115) or 125°C (K1-125), WH at 100°C for 6 min and expanded at 115°C (K2-115) or 125°C (K2-125), or WH at 100°C for 16 min and expanded at 115°C (K3-115) or 125°C (K3-125). Wet-heating and ET significantly (*P* < 0.001) increased the AME_n_ in FFSB. Among heat-treated FFSB, K1-115 and K1-125 resulted in the lowest and highest AME_n_ values, respectively, with all samples wet-heated at 100°C being intermediate. The K3-125 had AME_n_ values similar (*P* > 0.05) to K1-125. Among heat-treated FFSB, the highest average SID AA was recorded for K3-125. In Experiment 2, K3-125 from experiment 1 was divided into 9 batches and autoclaved at 110°C for 15 (Z1), 30 (Z2), 45 (Z3), 60 (Z4), 120 (Z5), 180 (Z6), 240 (Z7), 300 (Z8), and 360 (Z9) min. A quadratic (*P* < 0.01) pattern was observed for the effects of AT on AME_n_. The AME_n_ was unaffected until 300 min AT and then declined at 360 min. The AT quadratically (*P* < 0.001) affected the average SID AA where the SID increased from K3-125 to Z1, plateaued to Z5, and then declined to Z9. In conclusion, the results demonstrated that WH at 100°C for 16 min followed by expansion at 125°C as the most optimal wet-heating and expansion processing, associated with the highest SID AA. Autoclaving at 110°C for 30 min enhanced energy utilization and AA digestibility in FFSB, suggesting that further advantages may be achieved by short-time autoclaving of previously wet-heated and expanded FFSB samples.

## INTRODUCTION

Full-fat soybean (**FFSB**) is a valuable source of energy, protein, linoleic acid, vitamin E, and lecithin (Ravindran et al., 2014), and obtained by grinding whole soybeans without the removal of oil or cooking (Van Eys et al., 2004). However, due to the presence of several antinutritional factors (**ANFs**), more specifically trypsin inhibitors (**TI**; the Bowman-Birk Inhibitor and the Kunitz Inhibitor) and lectins, raw soybeans are not used in poultry diets (Liener, 1981; Waldroup, 1982; Grant, 1989). Trypsin inhibitors bind to proteolytic enzymes, forming inactive complexes and adversely affecting AA digestibility (Goebel and Stein, 2011). However, most of ANFs in soybeans are heat-labile, and therefore proper heat treatment will eliminate their adverse effects on AA digestibility and energy utilization. Properly processed FFSB can be used in broiler diets up to 200 to 250 g/kg without any adverse effects on performance (Waldroup et al., 1985; Leeson and Atteh, 1996; Clarke and Wiseman, 2007). However, Heger et al. (2016), reported that higher inclusions (340–400 g/kg) of a properly heat treated FFSB (expanded for 15 s at 125°C preceded by short-term [1 min] and long-term [5 min] conditioning at 100°C), resulted in similar performance to broilers (0–35 d) fed soybean meal-based diets.

The nutrient content and quality of FFSB is influenced by multiple factors including cultivar, agronomic and soil conditions, climate, and processing conditions. While the digestibility of AA in FFSB is influenced to a large extent by the adequacy of heat-treatment to inactivate the TI, both under- and over-processing are detrimental to the AA digestibility and energy utilization. The extent of heat-treatment is currently monitored in the industry through the in vitro laboratory tests such as urease activity (**UA**), protein solubility in 0.2% potassium hydroxide (**KOH**), protein dispersibility index (**PDI**), and trypsin inhibitor activity (**TIA**). Excessive heat processing of FFSB has been shown to result in low values for UA, KOH solubility, and PDI (Batal et al., 2000). Severe heat treatment can also damage the stability and digestibility of heat-labile AA such as Arg, Cys, and more specifically Lys, through the formation of Maillard reaction products (Pahm et al., 2008; Abdollahi et al., 2013).

The TIA of raw soybeans has been reported to be around 20 to 35 mg/g and it is vital to reduce the TIA to an industry recommended level of below 4.0 mg/g (Clarke and Wiseman, 2007) or between 1.75 and 2.50 mg/g (Van Eys et al., 2004; Ruiz, 2009). To eliminate the detrimental impacts of TI and to avoid excessive processing of FFSB, the hydrothermal processing needs to be optimised (Clarke and Wiseman, 2007). Several processing methods could be applied to heat treat raw soybeans and manufacture FFSB, which, depending on the degree of inactivation of ANFs or heat damage, can have different impacts on the nutritive value of FFSB (Ravindran et al., 2014). Among different thermal treatments, wet-heating, expansion, and extrusion, as the industry standards are commonly used to process the FFSB. Despite the potential and growing interest in the use of FFSB for poultry nutrition, only limited data are available on the effects of wet-heating (**WH**) and expanding characteristics on AA digestibility and apparent metabolizable energy (**AME**) of this feed ingredient (Kan et al., 1988; Valencia et al., 2009). Moreover, further research is warranted to investigate the potential of other processing techniques, such as autoclaving, to further optimize the hydrothermal processing of FFSB and its nutritional value. Autoclaving is a process that uses high-pressure (at least 103.4 kPa) saturated steam (up to 121°C) for about 15 to 20 min. This is an easily controllable process and widely utilized for disinfection and sterilization. Therefore, the objectives of the 2 studies reported herein were to assess the effects of 1) WH and expansion temperature (**ET**), and 2) autoclaving time (**AT**) on the chemical composition, protein quality indicators, AME, and standardized ileal digestibility (**SID**) of AA in FFSB.

## MATERIALS AND METHODS

### Heat Treatment of Full-Fat Soybeans

The influence of heat treatment on the metabolizable energy and SID AA of FFSB for broiler chickens was examined in 2 experiments. Experiment 1 was conducted to investigate the effects of short-time WH and ET on the AME and nitrogen-corrected AME (**AME_n_**) contents, and SID AA of FFSB. A batch of raw soybeans was obtained from Rieder Asamhof GmbH & Co. KG (Kissing, Germany) and used to develop 7 test FFSB samples. Prior to heat processing, the raw FFSB (K0) were initially mechanically cracked and processed further at the facilities of Amandus Kahl GmbH & Co. KG (Reinbek, Germany). The following equipments were used for the processing of FFSB used in Experiment 1: short-term conditioner (Amandus Kahl GmbH & Co. KG, Typ: DLM I), long-term conditioner (Amandus Kahl GmbH & Co. KG, Typ: LK 1605–2), and expander (Amandus Kahl GmbH & Co. KG, Typ: OEE 15.2). The conditioners were used for WH.

Prior to the expansion of FFSB, WH was required to prevent clogging of the screw during the expansion process. To produce K1, WH of raw FFSB (K0) was applied for 1 min at a temperature of 80°C, followed by 15 s expansion at either 115°C or 125°C to manufacture K1-115 and K1-125, respectively. The second batch of K0 was wet-heated at 100°C for 6 min (K2) and then expanded for 15 s at either 115°C (K2-115) or 125°C (K2-125). The final batch of K0 was wet-heated for 16 min at 100°C (K3), followed by 15 s expansion at either 115°C or 125°C to manufacture K3-115 and K3-125, respectively.

Experiment 2 was performed to investigate whether the AT, as an additional hydrothermal treatment, to previously wet-heated and expanded FFSB samples can further optimize heat treatment of FFSB (Herkelman et al., 1992), and benefit energy utilization and SID AA of FFSB for broiler chickens. For this purpose, a batch of K3-125 (wet-heated for 16 min at 100°C and expanded for 15 s at 125°C) sample was divided into 9 batches and autoclaved at 110°C for 15 (Z1), 30 (Z2), 45 (Z3), 60 (Z4), 120 (Z5), 180 (Z6), 240 (Z7), 300 (Z8), and 360 (Z9) min. The processing conditions for treatments in Experiments 1 and 2 are shown in Table 1. A fully automated autoclave (Typ HST 6 × 9 × 12, Zirbus Technology GmbH, Bad Grund, Germany) was used so that all settings of the processing conditions were defined in advance. Therefore, when the autoclave was filled with the FFSB samples, the process was controlled by the machine itself and according to the settings. Autoclaving enables to prolong the processing duration to the desired time, which is hard to achieve with other equipment generally used by the industry to process FFSB.

**Table 1:** Characteristics of heat treatment of full-fat soybeans.

		Wet-heating						Autoclaving								
		80°C		100°C				110°C								
	Raw	1 min	1 min	6 min	6 min	16 min	16 min	15 min	30 min	45 min	60 min	120 min	180 min	240 min	300 min	360 min
Heat treatment	K0	K1-115	K1-125	K2-115	K2-125	K3-115	K3-125	Z1	Z2	Z3	Z4	Z5	Z6	Z7	Z8	Z9
Conditioning at 80°C, min	-	1	1	-	-	-	-	-	-	-	-	-	-	-	-	-
Conditioning at 100°C, min	-	-	-	6	6	16	16	16	16	16	16	16	16	16	16	16
Expanding for 15 s, temperature^1^	-	115^2^	125^2^	115^3^	125^3^	115^3^	125^3^	125^3^	125^3^	125^3^	125^3^	125^3^	125^3^	125^3^	125^3^	125^3^
Autoclaving at 110°C, min^4^	-	-	-	-	-	-	-	15	30	45	60	120	180	240	300	360

1 The expander had a throughput of 1.5 to 1.8 t/h.

2 Specific energy inputs of 30.0 and 39.2-kilowatt hour/t were needed to reach expansion temperatures of 115 and 125°C, respectively.

3 Specific energy inputs of 13.1 and 20.0-kilowatt hour/t were needed to reach expansion temperatures of 115 and 125°C, respectively.

4 Steam pressure was set at 147 kPa. After heat treatment, all full-fat soybeans (excluding K0) were dried in a belt dryer at 85°C, and left the dryer after 5 min at 43°C. Dried samples were then transferred to the belt cooler and left it after 5 min at 30°C. Total of 7 samples were used in experiment 1: K0, K1-115, K1-125, K2-115, K2-125, K3-115 and K3-125. Total of 10 samples were used in experiment 2: K3-125, and Z1 to Z9.

### Assay Diets, Birds, and Housing

The experiments were conducted according to the New Zealand Revised Code of Ethical Conduct for the use of live animals for research, testing and teaching, and approved by the Massey University Animal Ethics Committee. Both experiments (Expt. 1, wet-heated and expanded samples; Expt. 2, autoclaved samples) were conducted as cohorts using the same research facility and consignment of birds.

### Determination of Apparent Metabolizable Energy

The AME of FFSB samples was determined by the difference method (Wu et al., 2020). In this method, a corn-soybean basal diet was formulated (Table 2) and 16 test diets, each containing different FFSB samples, were developed by replacing (w/w) 300 g/kg of the basal diet with one of the FFSB samples. The diets were offered in mash form.

**Table 2:** Composition (g/kg, as fed basis) of the basal diet used in the metabolizable energy assay^1^.

Item	Inclusion (g/kg)
Corn	610
Soybean meal	353
Dicalcium phosphate	22.0
Limestone	8.0
Sodium chloride	2.0
Sodium bicarbonate	2.0
Vitamin and trace mineral premix^2^	3.0

1 Analyzed protein content (N × 6.25) of the diet was 202 g/kg, as fed basis.

2 Supplied per kilogram of diet: antioxidant, 100 mg; biotin, 0.2 mg; calcium pantothenate, 12.8 mg; cholecalciferol, 60 µg; cyanocobalamin, 0.017 mg; folic acid, 5.2 mg; menadione, 4 mg; niacin, 35 mg; pyridoxine, 10 mg; trans-retinol, 3.33 mg; riboflavin, 12 mg; thiamine, 3.0 mg; dl-α-tocopheryl acetate, 60 mg; choline chloride, 638 mg; Co, 0.3 mg; Cu, 3.0 mg; Fe, 25 mg; I, 1 mg; Mn, 125 mg; Mo, 0.5 mg; Se, 200 µg; Zn, 60 mg.

Day-old male broiler chicks (Ross 308) were obtained from a commercial hatchery, raised in floor pens until 15 d of age and fed a commercial broiler starter diet (230 g/kg crude protein and 3,000 kcal/kg AME). The temperature was maintained at 31°C on d 1 and was gradually reduced to 22°C by the end of the third week. Central ceiling extraction fans and wall inlet ducts controlled the ventilation. The birds received 20-h of fluorescent illumination and allowed free access to the diets and water. On d 15, the birds were individually weighed, and 612 birds of uniform body weight were randomly allocated to 102 cages (6 birds per cage) with 6 replicates per treatment.

The AME was determined using the total excreta collection procedure from d 15 to 25. Diets were fed for 10 d (15–25 d post-hatch), with the first 6 d serving as an adaptation period. The feed intake and total excreta output for each replicate were recorded over the last 4 d of the assay. Daily excreta collections were pooled within replicates, mixed well in a blender and subsampled. Subsamples were lyophilized (Model 0610, Cuddon Engineering, Blenheim, New Zealand), and dried excreta samples were ground to pass through a 0.5-mm sieve and stored in airtight plastic containers at 4°C pending analysis. The diets and excreta samples were analyzed for dry matter (**DM**), gross energy (**GE**), and nitrogen (**N**).

### Determination of Standardized Ileal Digestibility of Amino Acids

A total of 16 assay diets containing different FFSB samples, as the only source of AA in the diet, and dextrose were formulated to contain about 180 g/kg dietary protein (Table 3; Ravindran et al., 2017). Basal endogenous AA losses (**EAA**) were measured using N-free diet (**NFD**) for the calculation of SID (Table 3). Titanium dioxide (3.0 g/kg; Merck KGaA, Darmstadt, Germany) was added to all diets as an indigestible marker. All diets were offered as mash.

**Table 3:** Composition of the test diets and nitrogen-free diet (NFD) (g/kg, as fed basis) used in the amino acid digestibility assay.

Item	Inclusion (g/kg)	
	Test diet	NFD
Full-fat soybean	500	-
Dextrose	441	870
Soybean oil	20.0	50.0
Dicalcium phosphate	19.0	19.0
Limestone	10.0	13.0
Titanium dioxide^1^	3.0	3.0
Sodium chloride	2.0	3.0
Sodium bicarbonate	2.0	3.0
Vitamin and trace mineral premix^2^	3.0	3.0
Cellulose^3^	-	35.0
Dipotassium phosphate	-	1.0

1 Merck KGaA, Darmstadt, Germany.

2 Supplied per kilogram of diet: antioxidant, 100 mg; biotin, 0.2 mg; calcium pantothenate, 12.8 mg; cholecalciferol, 60 µg; cyanocobalamin, 0.017 mg; folic acid, 5.2 mg; menadione, 4 mg; niacin, 35 mg; pyridoxine, 10 mg; trans-retinol, 3.33 mg; riboflavin, 12 mg; thiamine, 3.0 mg; dl-α-tocopheryl acetate, 60 mg; choline chloride, 638 mg; Co, 0.3 mg; Cu, 3.0 mg; Fe, 25 mg; I, 1 mg; Mn, 125 mg; Mo, 0.5 mg; Se, 200 µg; Zn, 60 mg.

3 Ceolus™, Microcrystalline Cellulose, Asahi Kasei Corporation, Tokyo, Japan.

Similar to the AME assay, both experiments were conducted as cohorts, using the same research facility and consignment of birds. Each of the 16 dietary treatments, plus the NFD, was offered to 6 cages (6 birds per cage) of male Ross 308 broilers. The test diets were offered for 3 d (from d 25 to 28), and birds had ad libitum access to test diets and water.

On d 28, all birds per cage, including those fed the NFD, were euthanized by intravenous injection (0.5 mL per kg body weight) of sodium pentobarbitone solution (Provet NZ Pty. Ltd., Auckland, New Zealand). Digesta were collected from the lower half of the ileum, as described by Ravindran et al. (2005). The ileum was considered as the portion of the small intestine from Meckel's diverticulum to a point about ∼40 mm proximal to the ileocecal junction. The ileal digesta were collected from all birds into a plastic container by gentle flushing with distilled water, pooled within a cage and stored at −20°C until lyophilized (Model 0610, Cuddon Engineering, Blenheim, New Zealand). The diet and freeze-dried digesta samples were ground to pass through a 0.5-mm sieve and stored in air-tight plastic containers at 4°C until the analysis of DM, titanium (Ti), N, and AA.

### Chemical Analysis

The DM was determined using the standard procedure (Method 930.15; AOAC International, 2016). Ether extract (**EE**), crude fiber, neutral detergent fiber (**NDF**), acid detergent fiber (**ADF**), sugars, and ash were analyzed according to methods described by Verband Deutscher Landwirtschaftlicher Untersuchungs und Forschungsanstalten (VDLUFA, 1976). Full-fat soybeans were analyzed for EE (method 5.1.1 using petroleum ether), crude fiber (method 6.1), NDF assayed with a heat-stable amylase and expressed inclusive of residual ash (method 6.5.1), ADF expressed inclusive of residual ash (method 6.5.2), sugars (method 7.1), and ash (method 8.1). Calcium and phosphorus were measured by Inductively Coupled Plasma-Optical Emission Spectroscopy (**ICP-OES**).

Gross energy was determined by adiabatic bomb calorimeter (Gallenkamp Autobomb, London, UK) standardized with benzoic acid. Nitrogen was analyzed by combustion (Method 968.06; AOAC International, 2016) using a CNS-200 carbon, N, sulfur analyzer (LECO Corporation, St. Joseph, MI). The crude protein (**CP**) content was calculated as N × 6.25. Titanium was measured on ultra-violet spectrophotometer following the method of Short et al. (1996). Content of reactive lysine (**rLys**) in the FFSB was determined as outlined by Fontaine et al. (2007), based on the transformation of Lys to its AA analog homoarginine by means of guanidination with o-methylisourea. The TIA was analyzed following method 71-10 (AACC 22-40.01, using DL-BAPA, 1995). The KOH solubility was determined as described by Araba and Dale (1990). The urease activity (**UA**) determination was based on the procedure of ISO 5506:1988 (1988), and the protein dispersibility index (**PDI**) was measured as outlined by AOCS (BA 10-65; 1980).

Amino acids were analyzed following standard procedures (Method 994.12; AOAC International, 2011). In brief, samples were hydrolyzed with 6 N HCl (containing phenol) for 24 h at 110 ± 2°C in glass tubes in an oven. The AA was measured using AA analyzer (ion exchange) with ninhydrin post column derivatization. The chromatograms were integrated using dedicated software (Agilent Open Lab software, Santa Clara, CA) with AA simultaneously detected at 570 and 440 nm. Cysteine and met were determined as cysteic acid and methionine sulphone, respectively, by oxidation with performic acid for 16 h at 0°C and neutralization with hydrobromic acid prior to hydrolysis. For Trp analysis, the samples were saponified under alkaline conditions with barium hydroxide octahydrate solution in the absence of air at 110°C for 20 h in an autoclave. Following alkaline hydrolysis, the internal standard α-methyl Trp was added to the mixture. After adjusting the hydrolysate to pH 3.0 and diluting with 30% methanol, Trp and the internal standard were separated by reverse phase chromatography on a HPLC column. Detection was selectively done with fluorescence detection (extinction 280 nm, emission 356 nm) to prevent interference by other AA and constituents.

### Calculations

All data were expressed on a DM basis, and the AME value of FFSB samples were calculated using the following formulas:

$$\text{AM}E_{\text{diet}}\left(\text{kcal}/\text{kg}\right)=\left[\left(\text{FI}\times GE_{\text{diet}}\right)-\left(\text{Excreta output}\times GE_{\text{excreta}}\right)\right]/\text{FI}$$

$$\text{AM}E_{\text{FFSB}}\left(\text{kcal}/\text{kg}\right)=\left[\text{AME of FFSB assay diet}-\left(\text{AME of basal diet}\times 0.70\right)\right]/0.30$$

Where, 0.70 = proportion of the basal diet in the assay diet, and 0.30 = proportion of FFSB sample in the assay diet.

Nitrogen retention, as a percentage of intake, was determined as follows:

$$N\;\text{retention }\left(\%\right)=100\times [((\text{FI}\times N_{\text{Diet}})-(\text{Excreta output}\times N_{\text{Excreta}}))/(\text{FI}\times N_{\text{Diet}})]$$

The AME_n_ was then calculated by correction for zero N retention by assuming 8.73 kcal per g N retained in the body as described by Titus et al. (1959).

$$N-\text{corrected AME }\left(\text{AM}E_{n};\;\text{kcal}/\text{kg}\right)=\;\text{AME}-\left(8.73\times N\;\text{retention}\right)/1000$$

The apparent ileal digestibility of AA was calculated from the dietary ratio of AA to Ti relative to the corresponding ratio in the ileal digesta using the following formula.

$$\text{AID of AA}=\left[\left[{\left(\text{AA}/\text{Ti}\right)}_{d}-{\left(\text{AA}/\text{Ti}\right)}_{i}\right]/{\left(\text{AA}/\text{Ti}\right)}_{d}\right]\times 100$$

Where, (AA/Ti)_d_ = ratio of AA to Ti in the diet, and (AA/Ti)_i_ = ratio of AA to Ti in the ileal digesta.

The basal EAA losses from birds fed the NFD were calculated as mg of AA flow per kg of DM intake (Moughan et al., 1992).

Basal EAA flow (mg/kg DM intake)=AA concentration in ileal digesta (mg/kg)×[diet Ti (mg/kg)/ileal digesta Ti (mg/kg)]

The apparent digestibility data for N and AA were standardized by using the basal EAA flow.

SID (%)=AID (%)+[basal EAA (mg/kg DM intake)/Ing. AA (mg/kg DM)]

Where, SID = standardized ileal digestibility of the AA; AID = apparent ileal digestibility of the AA; Basal EAA = basal endogenous AA loss, and Ing. AA = concentration of the AA in the ingredient.

### Statistical Analysis

In both experiments, data were analyzed by a one-way ANOVA using the GLM procedure of SAS (version 9.4; 2015; SAS Institute, Cary, NC.) in a completely randomized design. In experiment 2, orthogonal polynomial contrasts were performed to determine the linear and quadratic effects of autoclaving time. The cages were the experimental units. Differences were deemed significant when *P* ≤ 0.05 and, a *P*‐value between 0.05 and 0.10 was considered as a trend. The Least Significant Difference test was used to compare means.

## RESULTS

All birds remained healthy and readily consumed their assay diets throughout the study. No evidence of histopathological abnormalities was observed when the abdominal cavity was opened following euthanasia.

### Experiment 1 (Wet-Heating and Expansion)

The proximate, carbohydrate and mineral composition and in vitro protein quality indicators of the FFSB with different WH and ET are shown in Table 4. The DM, CP, EE, sugars, and minerals were similar among samples with different WH and ET, whereas the ADF, NDF, and CF contents of all heat-treated FSSB were lower than the raw sample (K0). The values of UA, PDI and TIA decreased in heat-treated samples compared to raw FFSB and the decline was greater beyond K1-115. The KOH protein solubility also reduced with heat-treatments severe than K1-115. The lowest values for UA, PDI, TIA, and KOH were determined in K3-125 samples. The WH and ET did not make any notable changes in CP, individual and total AA and rLys contents of FFSB samples (Table 5).

**Table 4:** Effect of heat treatment on the proximate, carbohydrate and mineral composition (g/kg, as received basis) and in vitro protein quality indicators of the 16 batches of full-fat soybeans (Experiments 1 and 2).

		Wet heating						Autoclaving								
		80°C		100°C				110°C								
	Raw	1 min	1 min	6 min	6 min	16 min	16 min	15 min	30 min	45 min	60 min	120 min	180 min	240 min	300 min	360 min
Item	K0	K1-115	K1-125	K2-115	K2-125	K3-115	K3-125	Z1	Z2	Z3	Z4	Z5	Z6	Z7	Z8	Z9
Dry matter	934	938	929	923	933	934	931	932	929	935	927	933	913	924	922	927
Crude protein	363	367	366	366	366	366	365	369	372	370	373	375	369	373	375	375
Ether extract	215	215	214	211	213	212	210	210	215	216	211	213	209	213	212	215
Sugars	93.0	99.2	89.0	93.7	78.9	92.0	81.9	91.1	93.4	88.8	85.6	88.8	78.5	74.8	68.5	61.8
Acid detergent fiber	83.3	62.5	60.4	70.4	68.4	75.7	62.1	69.9	69.7	61.0	68.2	70.6	82.8	77.6	99.7	127
Neutral detergent fiber	128	82.0	76.8	104	83.7	89.3	101	102	123	119	139	208	306	339	350	353
Crude fiber	78.0	42.3	49.2	54.8	45.9	58.2	54.5	51.1	55.2	41.4	54.1	60.5	65.8	58.4	63.1	61.0
Crude ash	54.3	55.0	53.1	54.8	54.2	56.4	51.9	52.8	52.9	52.5	52.5	54.4	51.6	52.8	54.2	55.5
Calcium	2.07	2.06	2.14	2.10	2.18	2.19	2.17	2.15	2.10	2.19	2.13	2.24	2.17	2.20	2.31	2.28
Phosphorus	5.91	5.98	6.07	6.01	5.91	5.95	5.93	5.96	5.91	6.03	5.97	6.01	5.83	6.11	6.04	6.23
Magnesium	2.14	2.19	2.24	2.23	2.23	2.21	2.19	2.20	2.16	2.18	2.16	2.23	2.19	2.25	2.19	2.27
UA, mg N_2_/g min	4.15	3.88	0.42	1.53	0.36	1.19	0.05	0.02	0.01	0.0	0.0	0.0	0.0	0.0	0.0	0.0
KOH, %	94.2	99.0	90.0	93.3	89.5	91.9	88.7	81.4	71.8	66.9	60.3	43.3	30.2	24.2	19.4	18.4
PDI, %	72.2	50.7	22.0	34.6	24.6	27.1	14.7	9.8	7.5	7.0	6.8	6.7	6.9	7.8	7.7	8.2
TIA, mg/g DM	27.3	24.0	7.2	14.4	5.9	8.7	5.3	2.4	1.5	1.0	0.8	0.5	0.4	0.4	0.3	0.2

Abbreviations: DM, dry matter; KOH, Protein solubility in 0.2% potassium hydroxide; PDI, protein dispersibility index; TIA, trypsin inhibitor activity; UA, urease activity.

**Table 5:** Analyzed amino acid (AA) contents (g/kg, as received basis) of 16 batches of full-fat soybeans (Experiments 1 and 2).

		Wet heating						Autoclaving								
		80°C		100°C				110°C								
	Raw	1 min	1 min	6 min	6 min	16 min	16 min	15 min	30 min	45 min	60 min	120 min	180 min	240 min	300 min	360 min
Item	K0	K1-115	K1-125	K2-115	K2-125	K3-115	K3-125	Z1	Z2	Z3	Z4	Z5	Z6	Z7	Z8	Z9
Crude protein	363	367	366	366	366	366	365	369	372	370	373	375	369	373	375	375
Indispensable AA																
Arg	26.4	26.8	26.9	26.7	26.6	26.2	26.5	26.7	26.1	25.8	25.9	25.1	23.7	22.6	21.4	19.5
His	9.41	9.55	9.54	9.54	9.52	9.37	9.45	9.56	9.38	9.36	9.44	9.30	9.24	9.19	9.10	8.81
Ile	16.2	16.4	16.3	16.3	16.2	15.7	16.3	16.2	16.6	16.4	16.4	16.3	16.6	16.7	16.8	16.7
Leu	27.5	27.9	27.8	27.7	27.6	27.3	27.7	27.8	27.6	27.4	27.5	27.9	27.4	27.8	27.8	27.7
Lys	22.7	23.1	23.0	22.9	22.9	22.5	22.8	22.5	22.0	21.7	21.5	20.4	18.7	17.5	16.3	14.7
rLys	20.9	21.4	21.2	21.0	21.2	20.7	21.1	20.5	20.0	19.5	19.0	16.8	14.4	12.4	10.6	8.87
rLys:Lys, %	92.4	92.9	92.1	91.4	92.6	91.7	92.8	91.0	90.9	89.9	88.4	82.6	76.9	70.6	65.0	60.3
Met	5.14	5.18	5.11	5.15	5.14	5.01	5.04	5.18	5.19	5.17	5.09	5.21	5.13	5.15	5.09	5.00
Phe	18.0	18.4	18.3	18.2	18.3	17.8	18.1	18.3	18.1	18.0	18.1	18.4	18.1	18.2	18.3	17.9
Thr	14.5	14.6	14.5	14.6	14.5	14.3	14.4	14.6	14.3	14.4	14.3	14.7	14.3	14.4	14.2	14.4
Trp	4.75	4.83	4.78	4.83	4.81	4.76	4.77	4.82	4.78	4.78	4.80	4.78	4.62	4.67	4.66	4.65
Val	17.0	17.5	17.2	17.2	17.1	16.6	17.1	17.1	17.6	17.3	17.4	17.0	17.4	17.6	17.6	17.5
Dispensable AA																
Ala	15.6	15.9	15.8	15.7	15.6	15.5	15.6	15.7	15.7	15.5	15.5	15.8	15.5	15.8	15.7	15.7
Asp	40.8	41.3	41.1	41.1	41.1	40.5	40.8	41.3	40.7	40.5	40.6	41.5	40.5	40.7	40.5	40.0
Cys^1^	5.51	5.85	5.90	5.87	5.81	5.75	5.76	5.60	5.44	5.35	5.20	5.03	4.61	4.46	4.29	4.04
Glu	63.5	64.6	64.3	64.1	64.0	63.2	63.8	64.5	64.0	63.8	63.9	65.2	63.9	64.7	64.6	63.8
Gly^1^	15.6	15.8	15.6	15.5	15.5	15.4	15.5	15.5	15.7	15.4	15.3	15.5	15.4	15.6	15.5	15.7
Pro	18.3	18.9	18.1	18.7	18.2	18.5	17.9	18.6	18.4	18.7	18.7	18.3	18.5	18.5	18.7	18.3
Ser	18.4	18.7	18.6	18.5	18.6	18.5	18.4	18.7	18.0	18.2	18.0	18.7	17.9	18.1	17.9	18.0
Total AA	339	345	343	342	341	337	340	343	339	338	338	339	331	332	328	322

Abbreviations: Ala, alanine; Arg, arginine; Asp, aspartic acid; Cys, cysteine; Glu, glutamic acid; Gly, glycine; His, histidine; Ile, isoleucine; Leu, leucine; Lys, lysine; Met, methionine; Phe, phenylalanine; Pro, proline; rLys, reactive lysine; Ser, serine; Thr, threonine; Trp, tryptophan; Val, valine.

1 Semi-indispensable amino acids for poultry.

The influence of WH and ET on the AME, AME_n_, and SID of protein and AA of FFSB in broilers is summarized in Table 6. Wet-heating and ET significantly (*P* < 0.001) increased the AME and AME_n_ in FFSB. The raw FFSB (K0) showed the lowest (*P* < 0.05) AME and AME_n_. Among the heat-treated samples, K1-115 (WH at 80°C for 1 min-expanded at 115°C) and K1-125 (WH at 80°C for 1 min-expanded at 125°C) resulted in the lowest and highest AME and AME_n_ values, respectively, with all samples wet-heated at 100°C being intermediate. However, the K3-125 sample (WH at 100°C for 16 min-expanded at 125°C) had AME and AME_n_ values similar (*P* > 0.05) to K1-125.

**Table 6:** The influence of wet-heating and expansion on the apparent metabolizable energy (AME), nitrogen-corrected AME (AME_n_), and standardized ileal digestibility^1^ (SID; %) of protein and amino acids (AA; %) of full-fat soybeans in broilers^2^ (Experiment 1).

		Wet-heating							
		80°C		100°C					
	Raw	1 min	1 min	6 min	6 min	16 min	16 min		
Item	K0	K1-115	K1-125	K2-115	K2-125	K3-115	K3-125	Pooled SEM	*P*-value
AME (kcal/kg DM)	1,634^d^	2,800^c^	3,523^a^	3,161^b^	3,295^b^	3,168^b^	3,370^ab^	76.7	0.001
AME_n_ (kcal/kg DM)	1,603^d^	2,649^c^	3,253^a^	2,917^b^	2,978^b^	2,917^b^	3,096^ab^	69.8	0.001
SID of protein	30.2^c^	54.9^b^	76.8^a^	69.9^a^	70.3^a^	71.1^a^	77.0^a^	2.50	0.001
SID of indispensable AA									
Arg	37.6^e^	63.1^d^	82.5^a^	74.1^c^	76.0^abc^	75.6^bc^	81.2^ab^	2.31	0.001
His	37.4^c^	57.8^b^	79.3^a^	73.8^a^	74.9^a^	74.2^a^	80.8^a^	2.52	0.001
Ile	17.0^d^	44.0^c^	73.8^ab^	68.4^b^	70.0^ab^	70.4^ab^	77.3^a^	2.56	0.001
Leu	20.0^d^	47.3^c^	74.8^ab^	69.0^b^	70.6^ab^	70.9^ab^	77.9^a^	2.55	0.001
Lys	42.2^d^	61.7^c^	81.4^a^	75.1^b^	76.6^ab^	76.4^ab^	82.1^a^	2.12	0.001
Met	29.7^d^	49.7^c^	77.8^ab^	74.5^b^	75.4^ab^	76.3^ab^	81.8^a^	2.38	0.001
Phe	20.4^d^	49.8^c^	76.3^ab^	69.3^b^	70.8^ab^	70.0^ab^	78.1^a^	2.84	0.001
Thr	25.1^c^	47.3^b^	72.3^a^	67.7^a^	68.6^a^	69.1^a^	74.6^a^	2.42	0.001
Trp	18.1^c^	41.5^b^	72.2^a^	70.2^a^	70.9^a^	71.6^a^	77.2^a^	2.74	0.001
Val	18.7^d^	44.5^c^	73.5^ab^	68.2^b^	69.8^ab^	70.2^ab^	76.8^a^	2.57	0.001
SID of dispensable AA									
Ala	26.4^d^	50.3^c^	75.5^ab^	69.7^b^	71.0^ab^	71.4^ab^	77.7^a^	2.50	0.001
Asp	27.4^d^	53.7^c^	77.2^a^	69.8^b^	71.6^ab^	70.1^b^	77.3^a^	2.39	0.001
Cys^3^	17.9^c^	32.9^b^	61.8^a^	59.0^a^	60.5^a^	59.6^a^	63.9^a^	3.03	0.001
Glu	37.0^d^	63.1^c^	81.9^a^	73.7^b^	74.4^b^	74.3^b^	81.0^a^	2.27	0.001
Gly^3^	25.0^d^	47.2^c^	72.8^ab^	67.4^b^	68.2^ab^	68.3^ab^	74.6^a^	2.51	0.001
Pro	38.4^d^	59.5^c^	79.5^ab^	74.7^b^	75.1^b^	76.2^ab^	81.4^a^	2.16	0.001
Ser	20.9^d^	47.5^c^	74.5^ab^	69.7^b^	71.0^ab^	70.6^ab^	77.8^a^	2.51	0.001
Average SID of all AA	27.0^d^	50.6^c^	75.7^ab^	70.3^b^	71.5^ab^	71.5^ab^	77.7^a^	2.43	0.001

Means in a row not sharing a common letter (a-e) are different (*P* < 0.05).

Abbreviations: Ala, alanine; Arg, arginine; Asp, aspartic acid; Cys, cysteine; Glu, glutamic acid; Gly, glycine; His, histidine; Ile, isoleucine; Leu, leucine; Lys, lysine; Met, methionine; Phe, phenylalanine; Pro, proline; Ser, serine; Thr, threonine; Trp, tryptophan; Val, valine.

1 Apparent digestibility values were standardized using the following basal ileal endogenous flow values (g/kg DM intake), determined by the feeding nitrogen-free diet: crude protein, 10.8; Arg, 0.44; His, 0.15; Ile, 0.37; Leu, 0.60; Lys, 0.39; Met, 0.15; Phe, 0.42; Thr, 0.52; Trp, 0.13; Val, 0.47; Ala, 0.40; Asp, 0.77; Cys, 0.22; Glu, 0.98; Gly, 0.42; Pro, 0.45; and Ser, 0.52.

2 Each value represents the mean of six replicates (six birds per replicate).

3 Semi-indispensable amino acids for poultry.

**Figure 1:**
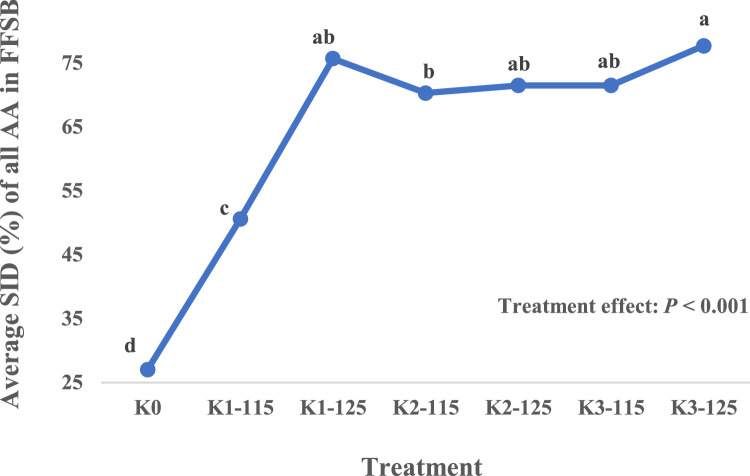
Average standardized ileal digestibility (SID) of all amino acids (AA) in full-fat soybeans (FFSB) in broilers as influenced by wet-heating and expansion temperature (Experiment 1). ^a-d^ Values with different superscripts differ significantly (*P* < 0.05).

A significant (*P* < 0.001) effect of WH and ET was observed on the SID of protein, all individual indispensable (**IAA**), dispensable (**DAA**) and average of all AA (Table 6). As could be expected, the raw FFSB (K0) resulted in strikingly lower (*P* < 0.05) SID protein and AA values. Among the heat-treated samples, the lowest SID AA was recorded for K1-115. Except the K1-115, all heat-treated samples showed similar (*P* > 0.05) SID for protein, His, Thr, Trp, and Cys. The K1-115 and K3-125 showed the lowest and highest SID of Ile, Leu, Met, Phe, Val, Ala, Gly, Pro, and Ser, respectively, with other heat-treated samples (K1-125, K2-115, K2-125, and K3-115) resulted in intermediate values. The K1-125 and K3-125 had similar (*P* > 0.05) SID values for Lys, Asp, and Glu, and higher (P < 0.05) than other heat-treated samples. For Arg, K1-125 had the greatest SID, followed by K3-125, K2-125, K3-115, and K2-115. Among heat-treated FFSB samples, the highest and lowest average SID of all AA were recorded for K3-125 and K1-115, with other samples being intermediate (Figure 1).

### Experiment 2 (Autoclaving Time)

The proximate, carbohydrate, and mineral composition and in vitro protein quality indicators of the FFSB autoclaved for different times are summarized in Table 4. The contents of DM, CP, EE, and minerals were similar among samples processed at different AT. The sugar content decreased at AT beyond 180 min. The ADF, NDF and CF contents increased when the AT was greater than 60 min. The values for UA, KOH protein solubility, PDI, and TIA decreased by autoclaving and the effect was higher with prolonged AT. Except for Arg, His, Lys, and Cys, the individual AA and protein contents remained similar among samples processed at different AT (Table 5). Contents of Arg, His, Lys, rLys, Cys, total AA, and the rLys:Lys ratio declined with increasing the AT beyond 60 min.

The influence of AT on the AME, AME_n_, and SID of protein and AA of FFSB in broilers is summarized in Table 7. The AT influenced the AME (*P* < 0.001) and AME_n_ (*P* < 0.05) of FFSB. Quadratic (*P* < 0.01) patterns were observed for the effects of AT on the AME and AME_n_. The AME content of FFSB was not influenced by AT until 180 min and declined at or above 240 min. The AME_n_, on the other hand, was unaffected until 300 min AT. A decline was observed only at 360 min AT.

**Table 7:** The influence of autoclaving time on the apparent metabolizable energy (AME), nitrogen-corrected AME (AME_n_), and standardized ileal digestibility^1^ (SID; %) of protein and amino acids (AA; %) of full-fat soybeans in broilers^2^ (Experiment 2).

	Autoclaving (110°C) time													
	0 min	15 min	30 min	45 min	60 min	120 min	180 min	240 min	300 min	360 min			Orthogonal polynomial contrasts	
Item	K3-125	Z1	Z2	Z3	Z4	Z5	Z6	Z7	Z8	Z9	SEM^3^	*P*-value	Linear	Quadratic
AME (kcal/kg DM)	3370^abc^	3547^a^	3520^a^	3476^ab^	3449^ab^	3357^abc^	3306^abc^	3214^bc^	3155^cd^	2914^d^	92.7	0.001	0.001	0.004
AME_n_ (kcal/kg DM)	3096^a^	3237^a^	3245^a^	3218^a^	3172^a^	3111^a^	3105^a^	3092^a^	3009^ab^	2793^b^	83.7	0.017	0.001	0.009
SID of protein	77.0^a^	81.2^a^	81.5^a^	79.3^ab^	80.7^a^	78.9^ab^	72.9^c^	67.0^d^	64.4^d^	51.2^e^	1.31	0.001	0.001	0.001
SID of indispensable AA														
Arg	81.2^b^	86.2^a^	87.1^a^	85.6^a^	86.3^a^	86.2^a^	81.6^b^	75.9^c^	73.4^c^	63.6^d^	1.21	0.001	0.001	0.001
His	80.8^b^	84.4^a^	84.9^a^	83.2^ab^	83.8^ab^	82.4^ab^	76.4^c^	71.0^d^	66.4^e^	55.7^f^	1.26	0.001	0.001	0.001
Ile	77.3^c^	82.2^ab^	83.4^a^	81.5^ab^	82.3^ab^	82.9^a^	78.7^bc^	70.9^d^	68.4^d^	56.5^e^	1.30	0.001	0.001	0.001
Leu	77.9^c^	82.7^ab^	84.0^ab^	82.8^ab^	83.6^ab^	84.7^a^	81.2^b^	74.7^cd^	71.5^d^	61.0^e^	1.16	0.001	0.001	0.001
Lys	82.1^ab^	85.3^a^	85.4^a^	82.7^a^	82.8^a^	78.6^b^	69.4^c^	61.2^d^	57.1^e^	40.2^f^	1.41	0.001	0.001	0.001
Met	81.8^bc^	86.1^a^	86.5^a^	84.4^ab^	84.9^ab^	84.4^ab^	78.9^c^	68.2^d^	65.7^d^	49.6^e^	1.48	0.001	0.001	0.001
Phe	78.1^cd^	82.5^ab^	84.0^ab^	83.1^ab^	83.8^ab^	84.6^a^	81.0^bc^	75.7^de^	73.0^e^	63.0^f^	1.18	0.001	0.001	0.001
Thr	74.6^ab^	78.8^a^	78.8^a^	75.7^a^	77.6^a^	75.9^a^	70.8^b^	63.2^c^	60.4^c^	47.7^d^	1.55	0.001	0.001	0.001
Trp	77.2^bc^	81.8^a^	82.5^a^	80.2^abc^	81.4^a^	80.7^ab^	76.3^c^	68.7^d^	64.8^d^	51.5^e^	1.38	0.001	0.001	0.001
Val	76.8^b^	81.8^a^	82.6^a^	80.6^ab^	81.8^a^	82.1^a^	77.6^b^	69.7^c^	66.9^c^	54.8^d^	1.35	0.001	0.001	0.001
SID of dispensable AA														
Ala	77.7^b^	82.0^a^	82.8^a^	81.3^a^	82.4^a^	82.0^a^	77.4^b^	70.6^c^	66.9^d^	55.0^e^	1.22	0.001	0.001	0.001
Asp	77.3^ab^	80.0^a^	79.0^ab^	75.3^b^	75.5^b^	69.4^c^	62.2^d^	56.3^e^	55.2^e^	41.8^f^	1.48	0.001	0.001	0.001
Cys^4^	63.9^a^	68.2^a^	66.6^a^	61.7^ab^	66.2^a^	57.1^b^	49.6^c^	44.2^cd^	41.2^d^	26.5^e^	2.37	0.001	0.001	0.001
Glu	81.0^c^	84.8^ab^	85.6^a^	83.6^abc^	84.0^abc^	82.1^bc^	76.8^d^	71.1^e^	69.0^e^	58.2^f^	1.19	0.001	0.001	0.001
Gly^4^	74.6^ab^	78.5^a^	78.4^ab^	75.4^ab^	77.1^ab^	74.1^b^	68.2^c^	61.9^d^	59.8^d^	47.1^e^	1.49	0.001	0.001	0.001
Pro	81.4^ab^	85.2^a^	85.8^a^	78.6^b^	79.8^b^	77.0^b^	71.8^c^	65.0^d^	63.0^d^	52.0^e^	1.56	0.001	0.001	0.001
Ser	77.8^bc^	81.7^a^	82.5^a^	81.2^ab^	81.5^a^	80.5^ab^	75.0^c^	68.7^d^	65.1^e^	52.9^f^	1.26	0.001	0.001	0.001
Average SID of all AA	77.7^b^	81.9^a^	82.3^a^	79.8^ab^	80.9^ab^	79.1^ab^	73.7^c^	66.9^d^	64.0^d^	51.6^e^	1.33	0.001	0.001	0.001

Means in a row not sharing a common letter (a-f) are different (*P* < 0.05).

Abbreviations: Ala, alanine; Asp, aspartic acid; Arg, arginine; Cys, cysteine; Glu, glutamic acid; Gly, glycine; His, histidine; Ile, isoleucine; Leu, leucine; Lys, lysine; Met, methionine; Phe, phenylalanine; Pro, proline; Ser, serine; Thr, threonine; Trp, tryptophan; Val, valine.

1 Apparent digestibility values were standardized using the following basal ileal endogenous flow values (g/kg DM intake), determined by the feeding nitrogen-free diet: crude protein, 10.8; Arg, 0.44; His, 0.15; Ile, 0.37; Leu, 0.60; Lys, 0.39; Met, 0.15; Phe, 0.42; Thr, 0.52; Trp, 0.13; Val, 0.47; Ala, 0.40; Asp, 0.77; Cys, 0.22; Glu, 0.98; Gly, 0.42; Pro, 0.45; and Ser, 0.52.

2 Each value represents the mean of six replicates (six birds per replicate).

3 Pooled SEM.

4 Semi-indispensable amino acids for poultry.

**Figure 2:**
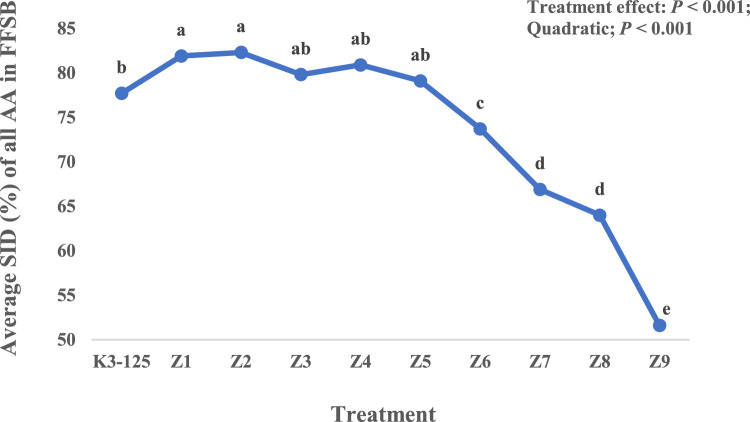
Average standardized ileal digestibility (SID) of all amino acids (AA) in full-fat soybeans (FFSB) in broilers as influenced by autoclaving time (Experiment 2). ^a-e^ Values with different superscripts differ significantly (*P* < 0.05).

Significant (*P* < 0.001) AT effects were recorded for the SID of protein and AA. The SID of protein remained unchanged by increasing AT from 0 (K3-125) to 120 min (Z5), and then declined resulting in a quadratic effect (*P* < 0.001). The AT quadratically (*P* < 0.001) affected the SID of all indispensable AA (except Lys and Thr), where the SID increased from 0 (K3-125) to 15 min (Z1) AT, plateaued up to 120 min (Z5), and then declined from 180 min (Z6) to 360 min (Z9) AT. Among dispensable AA, SID of Ala, Glu, and Ser increased from not-autoclaved sample (K3-125) to 15 min (Z1) AT, then plateaued, and followed by a reduction with AT above 120 min (Z6 to Z9). For Asp, Cys, and Gly, the SID remained constant from K3-125 to Z4 (60 min AT) and then declined with increasing AT above 60 min. The SID of Ser increased with autoclaving at 15 min (Z1), plateaued to 120 min (Z5), and then dropped as AT increased beyond 120 min. The AT quadratically (*P* < 0.001) affected the average SID of all AA where the SID increased from K3-125 to Z1, plateaued to 120 min (Z5) AT, and then declined to Z9 (360 min AT) (Figure 2).

## DISCUSSION

Published data on the effect of heat processing conditions, such as type, temperature, and duration of heat treatment, on the AME and SID AA of FFSB for poultry are scant (Kan et al., 1988; Valencia et al., 2009). Therefore, the paramount motive for this study was to investigate 1) whether wet-heating and different expansion temperature will influence AME and SID of AA in FFSB samples (Experiment 1), and 2) if the nutritional value of a previously heat-treated FFSB sample can be further improved by autoclaving at different times (Experiment 2).

### Experiment 1 (Wet-Heating and Expansion)

The proximate analysis, carbohydrate, and mineral composition of the raw FFSB were comparable to the values reported in the literature (Ravindran, 1988; Clarke and Wiseman, 2007; Thakur and Hurburgh, 2007; Valencia et al., 2009; NRC, 2012; Ravindran et al., 2014). Besides high protein content, substantial amount of EE exists in FFSB that were not influenced by WH and expansion, a finding that agrees with Kaankuka et al. (1996) and Kaewtapee et al. (2017). Although the data cannot be statistically compared, the WH and expansion seems to reduce the ADF, NDF, and CF contents of FFSB compared to the raw sample (K0), a finding that is not easy to explain.

Due to presence of TI, and their adverse effect on the digestibility of nutrients, especially of protein, the use of raw FFSB should be avoided in poultry diets (Waldroup, 1982; Grala et al., 1998). Although TI are heat-labile components and can be effectively eliminated through adequate heat treatment of raw FFSB, measures should be taken to avoid under- and over-heating and to ensure the protein quality of FFSB. Several in vitro protein quality indicators namely, UA index, KOH protein solubility test, PDI, and TIA, used by the industry to determine the extent of heat treatment and protein quality of soybean meal, have also been applied to FFSB (Ravindran et al., 2014), although their application to FFSB merits more investigation. An UA index between 0.00 and 0.10 pH unit change, KOH protein solubility of 78 to 85%, and PDI values between 15 and 30% have been suggested as current guidelines for adequately heat-treated soybean meals (Araba and Dale, 1990; Parsons et al., 1991; Van Eys et al., 2004; Ravindran et al., 2014). The current UA index is considerably below the earlier recommendation of 0.05 to 0.30 suggested by Hayward (1975). In the present study, even though all wet-heated and expanded samples had lower UA index compared to raw FFSB (K0), only WH at 100°C for 16 min and expanding at 125°C (K3-125) resulted in industry accepted recommendation for the UA index. The K1-125, K2-125, K3-115, and K3-125 samples were within the acceptable range of PDI (15–30%), however, only the K3-125 sample with PDI of 14.7% was close to the lower range limit of PDI. Although all the samples expanded at 125°C (K1-125; K2-125; K3-125) had lower KOH protein solubility than K0, the WH and expanding conditions investigated in Experiment 1 failed to meet the desired KOH protein solubility of 78 to 85%. Similarly, all wet-heated and expanded samples had TIA values well above the upper limit of 2.5 mg TIA/g of soybean meal (Ruiz, 2009). TIA values of below 4.0 mg/g FFSB (Clarke and Wiseman, 2007) and of 1.75 to 2.50 mg/g soybean meal are considered necessary to mitigate the negative impact of TI on AA digestibility (Van Eys et al., 2004; Ruiz, 2009). These findings indicate some degree of under-processing and the potential for further improvement of the nutritional quality of FFSB by additional processing techniques such as autoclaving.

The AA composition of raw FFSB in the current study was within the range previously reported (Valencia et al., 2009; NRC, 2012; Ravindran et al., 2014), and remained unchanged following WH and expansion, highlighting the fact that heat treatment mainly influences digestibility of nutrients (Wiseman, 2013) with minimal impact on the content of nutrients. High temperature and low moisture contents used in thermo-mechanical treatments can favor the formation of Maillard reaction products (Voragen et al., 1995; Thomas et al., 1998; Fontaine et al., 2007). Free aldehyde groups from reducing sugars and free amino groups from AA, the epsilon-amino group in particular, can interact and result in the destruction of some AA, specifically Lys that can be characterized by the reduction of rLys (Fontaine et al., 2007). Intensified heat treatments may also result in marked degradation of Cys, the most heat-labile AA, followed by Lys and, loss of Arg, Thr, and Ser (Papadopoulos, 1989). The lack of WH and expansion effect on the concentration of rLys, rLys:Lys ratio, and Cys in our study may indicate that the heat processing conditions applied were not severe to damage these AA.

In the present study, WH and expansion, regardless of temperature, of FFSB enhanced the AME and AME_n_ content over raw FFSB. The highest AME and AME_n_ values were determined for K1-125, followed by the K3-125 sample. Compared to the raw sample, K1-125 and K3-125 samples supported a higher AME_n_ by 103% (3,253 vs. 1,603 kcal/kg), and by 93% (3,096 vs. 1,603 kcal/kg).

Kan et al. (1988) reported that dry pelleting increased the metabolizable energy content of FFSB by 383 kcal/kg (from 3,271 to 3,654 kcal/kg) in broilers, but no information was given on the pelleting process conditions. The improvement in AME_n_ was attributed to an increase in fat digestibility of FFSB from 72 to 88% following dry pelleting. The high fat content and high lipid digestibility ostensibly contribute to the AME_n_ of FFSB. These observations suggest that proper heat treatment, WH at 80°C for 1 min and expansion at 125°C for 15 s, as applied in the current study, may release encapsulated lipids within the cell wall and enhance fat digestion and that energy utilization is advantaged consequently.

Except the K1-125 and K3-125 samples, digestibility values for protein and individual AA determined in the current work were lower than those reported for micronized FFSB (Valencia et al., 2009) and Ravindran et al. (2014). Among all processing conditions in the current study, WH at 100°C for 16 min and expanding at 125°C (K3-125) resulted in the highest SID of CP and AA, followed by K1-125 (WH at 80°C for 1 min and expanding at 125°C). The lowest SID of protein, individual AA, and average SID of all AA was observed in K0 followed by K1-115, confirming that raw FFSB is an unsuitable ingredient for use in poultry diets (Waldroup, 1982) and, short time (1 min) WH at 80°C prior to expansion at 115°C for 15 s is inadequate to improve the SID of CP and AA of FFSB. Similar findings have been reported for AA digestibility of FFSB in pigs, where FFSM sample wet-heated at 80°C prior to 15-s expansion at 125°C showed lower SID of CP and AA compared to those with WH at 100°C and expanded at 125°C for 15 s (Kaewtapee et al., 2017). The highest average SID of all AA observed in K3-125 sample (Figure 1) which corresponded to the lowest values for all in vitro protein quality indicators, suggesting WH at 100°C for 16 min followed by 15-s expansion at 125°C as the most optimal heat treatment of FFSB. Even though the heat treatments reduced the TIA, especially in those beyond K1-115 and the K3-125 sample containing the lowest TIA, the presence of TI might not be the sole factor for the lower AA digestibility in raw or under-processed FFSB samples. Kakade et al. (1973), by removing the TI from an extract of raw soybeans, reported that only 40% of the improvement in protein utilization of raw soybeans achieved by heat treatment could be attributed to the removal of TI. Similar to the current findings, Leeson and Atteh (1996) reported a reduction in TIA from 58.7 mg/g in raw soybeans to 16.1, 14.8, 9.3, and 8.4 mg/g following the extrusion of soybeans at 80, 100, 120, and 140°C, respectively. However, extrusion temperature failed to have any significant effect on retention of nitrogen, fat, Ca, and P in broilers, suggesting factors other than TIA contribute to the digestibility of nutrients in heat-treated soybeans (Leeson and Atteh, 1996). Therefore, the increases of SID AA in heat-treated FFSB samples in the present study could be explained by combination of amelioration of the detrimental effects of TI and, though not measured, by partial denaturation of the raw protein prior to digestion (Liener, 1976; Hans and Parsons, 1991), and elimination of the antinutrient effects of lectins (Smith and Circle, 1972).

### Experiment 2 (Autoclaving Time)

In a quest to achieve even more satisfactory processing, the FFSB sample with the most optimal in vitro protein quality indicators and the highest SID AA (K3-125; WH at 100°C for 16 min followed by 15-s expansion at 125°C) in Experiment 1 was subjected to additional hydrothermal treatment in the form of autoclaving at 110°C for different durations. Autoclaving further reduced the UA, KOH protein solubility, PDI and TIA, with greater impact as AT increased. A gradual decline in KOH protein solubility from 88.7% to 18.4%, and TIA from 5.3 to 0.2 mg/g observed from K3-125 to the longest autoclaving time (Z9). Similarly, Clarke and Wiseman (2007) reported a reduction in TIA of FFSB form 28.4 mg/g in raw soybeans to 14.8, 9.6, 4.5, and 1.9 mg/g following extrusion at 90, 110, 130, and 160°C. However, in the current study, the reduction in UA and PDI was recorded only up to 45 min autoclaving, with no further major reduction after this point. Urease test is an indirect test of TIA, based on the assumption that the heat denatures TI and urease to a similar extent, and a high correlation has been reported between the urease index and TIA, confirming the validity of this assumption (Ravindran et al., 2014). However, the results of the present experiment agree with previous reports that UA test, while a useful indicator of adequate heating to deactivate the antinutritional factors, is not a good indicator to assess whether the soybean product has undergone an excessive heat treatment beyond the threshold of urease inactivation. Therefore, UA test should not be used as the sole measure of optimum soybean processing (Abraham et al., 1971; Araba and Dale, 1990). In Experiment 2, TIA of 1.5 to 2.4 mg/g recorded for Z1 and Z2 samples was associated with the highest average SID of all AA (Figure 2). In agreement, Clarke and Wiseman (2007) measured the digestibility of some AA in FFSB samples with different TIA contents of 14.8, 9.6, 4.5, and 1.9 mg/g with the highest AA digestibility recorded in FFSB sample with 1.9 mg TIA/g.

The reduction in concentrations of Arg, His, Cys, Lys, rLys, and the rLys:Lys ratio with increasing AT, especially beyond 60 min, can be indicative of the initial point of over-processing. While the rLys content decreased by 10% from 21.1 g/kg in K3-125 to 19.0 g/kg in Z4, a severe decline of 58% was recorded when the AT increased from 0 to 360 min (21.1 g/kg in K3-125 vs. 8.87 g/kg in Z9). Interestingly, the sugar content of autoclaved samples reduced with prolonged AT, which, although not conclusive, might be an indirect indicator of the formation of Maillard reaction products (Voragen et al., 1995; Thomas et al., 1998; Fontaine et al., 2007), from reducing sugars and the epsilon-amino group of Lys, with the consequent reduction of rLys (Fontaine et al., 2007).

In Experiment 2, the AME content of FFSB was impaired with autoclaving time at or above 240 min. However, the AME_n_ declined only at 360 min AT. In general, when the previously wet-heated and expanded FFSB samples (K3-125) were autoclaved at 110°C for 30 min (Z2), the AME and AME_n_ increased by 150 kcal/kg over K3-125 sample. However, this advantage was eroded from 150 kcal/kg in Z2 to 79 (AME) and 76 (AME_n_) kcal/kg in Z4 (60 min AT), and was transformed into a disadvantage of −156 kcal AME/kg (3,214 vs. 3,370) in Z7 (240 min AT) and -303 kcal AME_n_/kg (2793 versus 3370) in Z9 (360 min AT). It is tempting to suggest that the AME, rather than AME_n_, responses should be considered when evaluating the optimum heat processing of FFSB. This is because correcting the AME to zero N retention (AME_n_) penalizes more severely the energy value of properly heat treated FFSB samples, with higher protein quality and N retention, compared to those under or over processed, and consequently evens out the differences between AME_n_ of differently processed FFSB samples; a scenario that also applies to other protein sources (Abdollahi et al., 2021).

Similar to the findings on AME and AME_n,_ autoclaving at 110°C for 30 min (Z2), increased the average SID of all AA by 5.92% compared to the K3-125 sample (Figure 2), but this advantage was progressively diminished from 5.92% in Z2 (82.3 vs. 77.7%) to only 2.70% when the AT increased by 15 min in Z3 (79.8 versus 77.7%). These benefits were transformed into disadvantages in the average SID of all AA of −5.15, −13.9, −17.6, and −33.6% in Z6, Z7, Z8, and Z9, respectively. The magnitude of the AA digestibility reductions because of prolonged AT were inconsistent across different AA. Among all AA, the greatest decline in SID AA of Z9 sample, compared to K3-125, was recorded for Cys (59%), followed by Lys (51%), Asp (46%), Met (39%), Gly (37%), Thr and Pro (36%), Trp (33%), Ser (32%), His (31%), Val and Ala (29%), Glu (28%), Ile (27%), Arg and Leu (22%), and Phe (19%). These findings are in general agreement with previous reports identifying Cys, Lys, Arg, Thr, and Ser as the most heat-labile AA (Papadopoulos, 1989). However, it should be recognized that heat-induced structural damages to an AA might not be accurately captured by digestibility measurements since the damaged AA can be digested and absorbed without the ability to participate in metabolic reactions in the animal body (Häffner et al., 2000).

One implication of this work is that while autoclaving of properly wet-heated and expanded FFSB samples has the potential to enhance energy utilization and AA digestibility of FFSB, they need not be autoclaved for prolonged times. More benefits from autoclaving could be realized with AT of up to 30 min. Compared to industry recommendations for heat-processed soybean meal and supported by the enhanced energy utilization and digestibility of AA in FFSB autoclaved at 110°C for 30 min (Z2) and, while not conclusive, it is plausible to suggest that different, and a slightly lower range of, in vitro protein quality indicators may need to be used to screen heat-treated FFSB samples. The UA of 0.00 to 0.02 pH units, KOH protein solubility of 72 to 81%, PDI of 7.5 to 10%, and TIA of 1.5 to 2.4 mg/kg could be the appropriate in vitro protein quality indicators for FFSB. However, identifying the optimum in vitro protein quality indicators to be used in poultry industry will require further research investigating different thermal treatments.

## CONCLUSIONS

The results of Experiment 1 demonstrated WH at 100°C for 16 min followed by 15-s expansion at 125°C as the most optimal wet-heating and expansion processing, resulting in optimum in vitro protein quality indicators associated with the highest SID AA. The data from Experiment 2 suggest that FFSB used in the poultry industry might not be satisfactorily processed and that further short-time autoclaving of the previously wet-heated and expanded FFSB samples would be more desirable than complete reliance on wet heating and expansion. Autoclaving at 110°C for 30 min enhanced energy utilization and AA digestibility in FFSB, suggesting that additional advantages may be achieved by autoclaving FFSB to a TIA content of 1.5 mg/g. Finally, the current study provided further evidence that prolonged autoclaving of FFSB, beyond 30 min, to inactivate TI can deteriorate the digestibility of AA, especially of Cys and Lys, and must be avoided.
